# Technology-Driven Group Exercise Program Implementation in an Underserved Community: Multimethod Retrospective Evaluation Study

**DOI:** 10.2196/79598

**Published:** 2026-03-24

**Authors:** Whitney N Neal, Laurie A Malone

**Affiliations:** 1 Department of Health Behavior, School of Public Health, University of Alabama at Birmingham, 1665 University Boulevard, Birmingham, 35294, United States, +1 (205) 996-0825; 2 Department of Occupational Therapy, School of Health Professions, University of Alabama at Birmingham, Birmingham, AL, United States

**Keywords:** physical activity, underserved communities, group fitness intervention, program implementation, exercise

## Abstract

**Background:**

Physical inactivity among people with disabilities and older adults is a persistent public health concern, particularly those from racial minority groups living in underserved communities where structural barriers limit access to exercise opportunities. Technology-driven exercise programs offer scalable solutions, but the contextual factors that influence their uptake, fidelity, and sustainability remain underexplored.

**Objective:**

This study applied the Consolidated Framework for Implementation Research (CFIR) to identify barriers, facilitators, and lessons learned during the implementation of 2 technology-driven group exercise programs—synchronous online fitness and active virtual reality gaming—delivered to predominantly African American older adults with disabilities and/or chronic health conditions in an underserved community.

**Methods:**

Using a multimethod design, we conducted semistructured interviews and focus groups with program participants, staff, and administrators (n=15) and administered implementation surveys to program deliverers (n=9). Qualitative data were analyzed deductively using CFIR domains and constructs, while survey data provided descriptive context.

**Results:**

Both programs achieved high acceptability and appropriateness scores, with feasibility rated slightly lower for virtual reality. Themes mapped to 4 CFIR domains (innovation, outer setting, inner setting, and individuals). Qualitative analysis identified adaptability, affordability, engaging design, and community support as key facilitators, while barriers included technology and space constraints, resistance to new technology, and sustainability challenges.

**Conclusions:**

Technology-driven exercise programs can expand access to physical activity in underserved communities when they are adaptable, affordable, and socially engaging. Addressing multilevel barriers, including resource limitations, technology hesitancy, and long-term sustainability, is critical to scaling and sustaining these interventions.

## Introduction

Despite the known health benefits of being physically active, people with disabilities report high levels of physical inactivity. Reports worldwide indicate that approximately 54% to 91% of adults without disabilities meet physical activity recommendations, compared with only 21% to 60% of people with disabilities [1]. In the United States, 1 in 4 adults is living with a disability [2], and sedentary people with disabilities are 3 times more likely to report primary health conditions such as heart disease, stroke, diabetes, or cancer compared to active people with disabilities [3]. Furthermore, physical inactivity exacerbates loneliness and social isolation, which are also associated with adverse health outcomes, including heart disease and stroke [4]. Of concern is that individuals with disabilities are not only physically inactive but often experience higher levels of social isolation and loneliness than persons without disabilities [5]. Together, these disparities underscore the urgent need for effective, inclusive strategies to promote physical activity among people with disabilities.

Barriers to physical activity for people with disabilities span multiple levels of the socioecological model, including individual (eg, lack of knowledge, physical limitations, pain, and fatigue), interpersonal (eg, lack of social support), institutional or organizational (eg, lack of accessible fitness facilities and exercise equipment), community (eg, poorly designed outdoor spaces and street connectivity), and systemic (eg, limited or no transportation) factors [1,6-10]. These multifactorial barriers limit opportunities for people with disabilities to engage in effective and sustainable physical activity and are further compounded in underserved communities—those characterized by racial and ethnic health disparities, high poverty rates, low insurance coverage, and limited health care and physical activity resources—underscoring the need to address physical activity barriers within the socioecological framework [11,12].

Technology-driven physical activity [13] and health promotion programming can help eliminate many of these barriers by delivering accessible, scalable interventions that foster social engagement and a sense of community [14-17]. Recent systematic reviews and meta-analyses demonstrate that mobile and wearable technologies, telehealth-delivered exercise, online fitness programs, and interactive gaming platforms can increase physical activity levels and improve body composition and physical function, with consistent benefits shown across home-based and community settings [18-20]. Telehealth programs have shown improvements in physical functioning even among individuals with frailty, cognitive challenges, or mobility limitations, highlighting the value of remote delivery for populations facing barriers to traditional exercise access [21]. Adherence to technology-based exercise is generally favorable when programs are accessible, engaging, and designed to meet participant needs [22]. Interactive and game-based technologies further extend reach and appeal: virtual reality (VR) exergame programs have been shown to improve balance, mobility, and functional outcomes in older adults, including those living in long-term care, while enhancing enjoyment and motivation to be active [23-25]. Work by Malone et al [15,26] demonstrates that adults with mobility impairments can engage with adapted exergaming systems—such as modified Wii Fit balance boards and gaming mats—to achieve greater energy expenditure and report high enjoyment, suggesting these technologies can reduce barriers and support participation in meaningful movement. Additionally, online membership–based fitness programs have shown feasibility and preliminary effectiveness for increasing physical activity in people with mobility impairments [27]. Collectively, this body of evidence supports the use of technology-driven physical activity interventions as accessible, engaging, and adaptable options for promoting physical activity and supporting health in populations traditionally underserved by conventional exercise models.

Although technology-mediated exercise shows promise, evidence of efficacy and feasibility alone is insufficient to ensure successful deployment in resource-limited settings. To translate and sustain such programs in underserved settings, it is essential to examine multilevel determinants of implementation. Implementation science offers a structured approach to understanding and addressing contextual factors that influence the uptake, fidelity, and sustainability of health interventions [28]. The Consolidated Framework for Implementation Research (CFIR) provides a comprehensive, validated taxonomy for assessing contextual factors that influence implementation success across 5 domains: outer setting, inner setting, innovation, implementation process, and individuals [29,30]. Using a framework like the CFIR ensures that barriers and facilitators are identified and addressed in a structured, reproducible manner, strengthening both the science and practice of health promotion program delivery in underserved settings. The CFIR has been used to identify implementation barriers and facilitators of physical activity interventions for older adults and people with disabilities [31-33], and in a systematic review of community-based physical activity interventions [34]. However, research on technology-driven exercise interventions in underserved communities remains limited.

To address this gap, this study used the CFIR to identify facilitators, barriers, and lessons learned during the implementation of 2 technology-driven group exercise programs (synchronous online fitness and VR exergaming) delivered to older adults (aged >60 y) and individuals with disabilities and/or chronic health conditions living in an underserved community. Our primary aims were to (1) characterize the CFIR constructs that most strongly influenced each program’s implementation outcomes and (2) compare implementation team member perspectives (program staff, administrators, and participants) on technology-specific and context-specific challenges and enablers. By leveraging a validated implementation framework, this work examines how technological and contextual factors shape program delivery in underserved communities and informs strategies to translate and sustain effective technology-based exercise in traditionally under-resourced settings.

## Methods

### Study Design and Setting

#### Overview

This study followed a multimethod research design, whereby the core of the research was qualitative, utilizing in-depth interviews and focus groups, and was supplemented by a minor quantitative component in the form of a brief survey provided to program staff and administrators. We chose both methods not for the purpose of triangulation or formal mixing of qualitative and quantitative paradigms, but rather for complementarity and expansion [35], allowing the survey data to offer a broader, descriptive overview of factors associated with the Lakeshore Online Fitness (LOF) and VR program implementation and the rich qualitative data to provide deep context.

**Figure 1.**
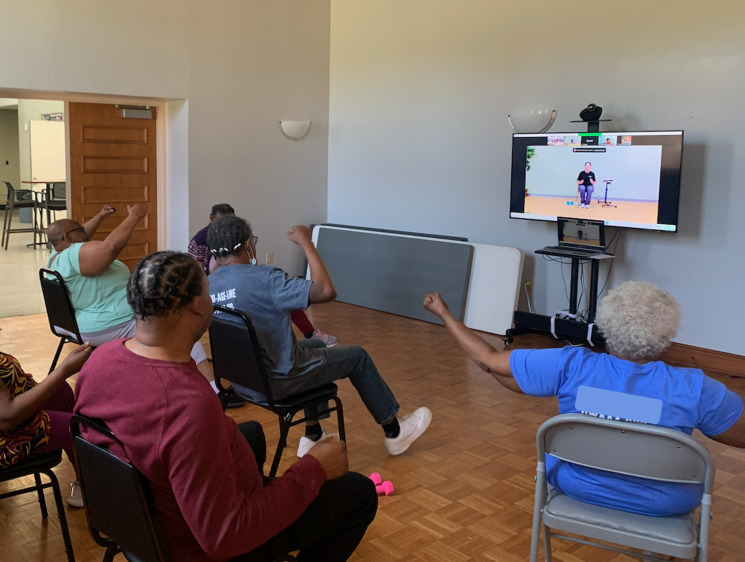
Participants engaging in the “Lakeshore Online Fitness” program.

**Figure 2.**
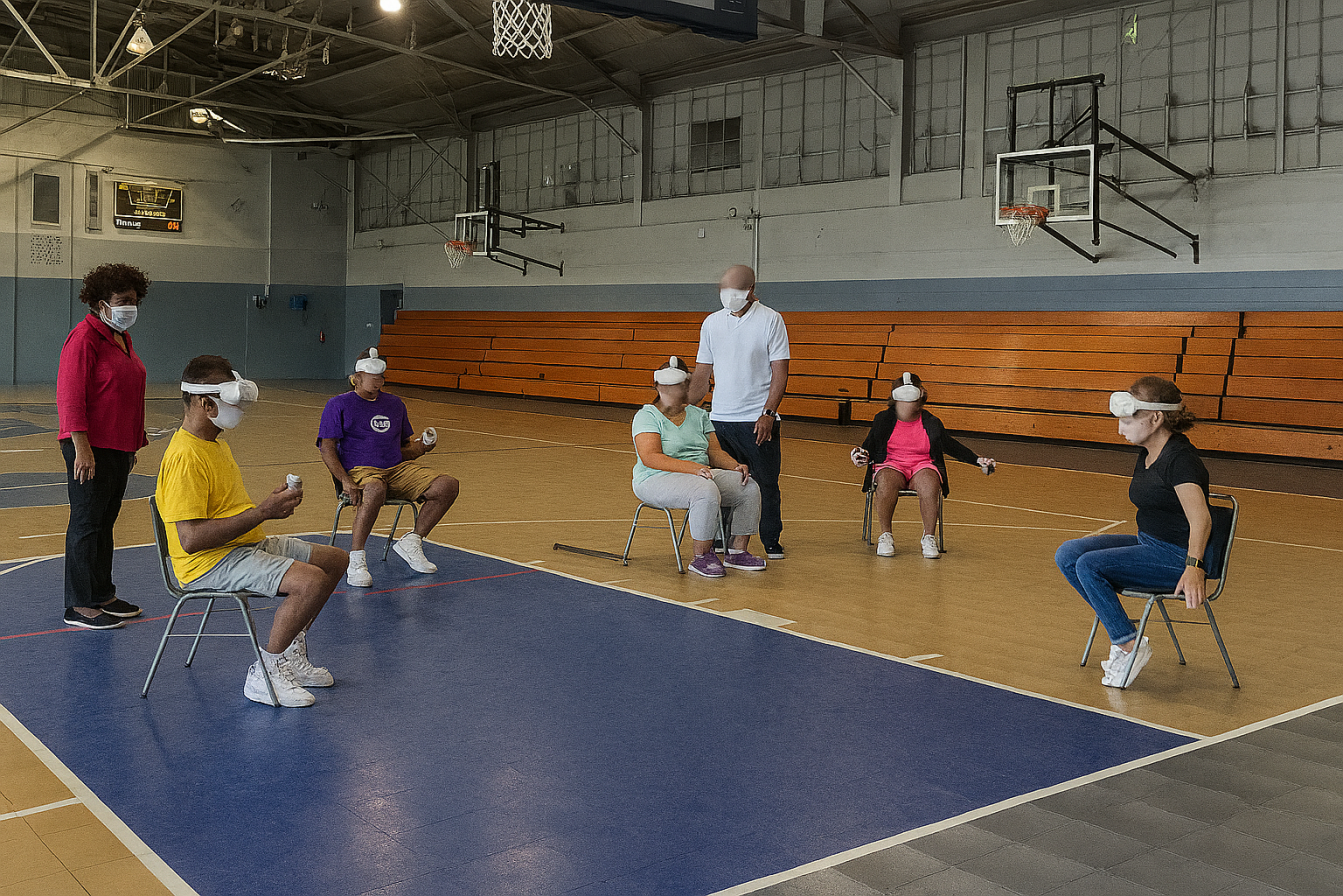
Participants engaging in the “Get Active with Virtual Reality” program.

This research was a retrospective evaluation of 2 technology-based interventions deployed between July 2022 and October 2022 at a neighborhood recreation center located in an underserved community (population <5800; ≈50% Black, ≈15% Hispanic or Latino; ≥25% living below the federal poverty line; 1 in 4 uninsured; 1 in 4 with a disability). The synchronous online fitness program was deployed in partnership with a local nonprofit organization that provides physical activity, recreation, and sport programs for people with physical disabilities and chronic health conditions (Lakeshore Foundation). For the VR program component, content- and age-appropriate (ie, limited violence or foul language) active video games were identified by research staff. The LOF and Get Active with VR programs were delivered on-site at the municipal recreation facility building. Program recipients were current members of the recreation center’s senior programs who met the following eligibility criteria: (1) aged 18 years or older, (2) self-reported a physical disability or health condition, (3) aged 60 years or older regardless of health condition, and (4) were not enrolled in any other physical activity program. All successfully screened participants provided informed consent prior to enrollment. Sessions for both programs were held twice a week, lasted 60 minutes, and included a warm-up, main activity, and cooldown. Participants completed 6 weeks of each program (LOF or VR) in a randomized crossover design using alternating assignments to either the LOF (Figure 1) or VR program (Figure 2).

#### Lakeshore Online Fitness

Online classes were delivered via Zoom (Zoom Communications, Inc) from Lakeshore to the municipal recreation facility [27,36]. Exercise dose was based on the predetermined length of online classes provided by Lakeshore, and frequency was based on scheduling needs at the recreation center. An instructor from Lakeshore taught all classes, and a community facilitator (staff or volunteer) and research team member oversaw the on-site group activity.

#### Get Active With Virtual Reality

Oculus Quest 2 headsets were used for the VR program. A community facilitator and research team member oversaw VR classes. For each session, program recipients would choose to play a predetermined game individually or competitively against other participants. Exercise dose and frequency were matched to those of the LOF program.

### Study Participants

This research study used convenience sampling techniques to identify factors related to program implementation. Our sample size was determined by the availability of respondents and met minimum guidelines for thematic analysis [37,38]. We gathered input from individuals who (1) participated in the LOF and/or VR programs and (2) played a role in program implementation and/or delivery. Invitations for interviews were extended to program recipients who completed the LOF and VR programs (n=10). Invitations for focus groups were extended to 2 LOF instructors, 2 Lakeshore Foundation administrators, 1 recreation center administrator, 1 recreation center program staff member, and 2 community volunteers (n=9). The recreation center staff member’s primary role was to help with the physical setup for LOF, assist participants during classes, and take down program equipment. Both community volunteers were recruited through the University of Alabama at Birmingham Center for the Study of Community Health to act as community liaisons, assist with classes, and identify potential new locations for implementation of the programs. These volunteers also helped with the physical setup for the VR program, assisted participants during classes, and took down program equipment.

### Ethical Considerations

The University of Alabama at Birmingham Institutional Review Board approved this study (IRB-300008762), and all program participants provided written informed consent before taking part, which included agreement to participate in the study and permission for their images to appear in photos used in the study. Program participants received US $15 for completing the interviews. Data were stored on encrypted, password-protected servers accessible only to the research team.

### Data Collection

#### Quantitative

Administrators, program staff, and volunteers involved in implementing the LOF and VR programs were invited to complete an online survey assessing accessibility, appropriateness, and feasibility of the two programs in October 2023. The Acceptability of Intervention Measure (AIM), Intervention Appropriateness Measure (IAM), and Feasibility of Intervention Measure (FIM) surveys were completed by all 9 relevant staff members and administrators. The AIM assesses the “perception that a given treatment, service, practice, or innovation is aggregable, palatable, or satisfactory,” the IAM evaluates the “perceived fit of the innovation to address a particular issue or problem,” while the FIM measures the ease at which a “new treatment or innovation can be successfully used or carried out within a given agency or setting.” [39] Each measure consisted of 4 thematic questions, with 5 responses ranging from completely disagree to completely agree on a 5-point Likert scale [40]. Outcomes for the LOF and VR programs were assessed separately. Scores for each scale range from 4 to 20 and were classified as low (<12), medium (12-15), and high (>15) [40].

#### Qualitative

Program participants took part in one-on-one, semistructured interviews at the recreation center in January 2023 after completing the intervention. A CFIR-informed interview guide, developed in collaboration with the study principal investigator (LAM) and interviewer (WNN), was used to gauge perceptions of the most important factors influencing implementation of the 2 programs for older adults and people with disabilities in underserved communities. Interview guide questions focused on the individual, innovation, and inner setting domains of the CFIR (Multimedia Appendix 1) [29].

A separate CFIR-informed focus group guide was developed to capture staff members’ opinions about barriers, facilitators, and lessons learned from implementation of the programs (Multimedia Appendix 2). Focus group questions covered the innovation, outer setting, inner setting, and implementation process domains of the CFIR [29]. Two semistructured, in-depth focus groups and 1 interview were carried out. The first focus group (n=3) included the recreation center program staff member and two community volunteers who helped set up and deliver the LOF and VR programs. The second focus group (n=3) comprised LOF fitness instructors who delivered the LOF program and 1 Lakeshore Foundation senior staff member. The focus group with recreation center staff and volunteers was conducted in person at the community center where participants completed the programs, while the focus group with LOF program deliverers and senior staff was carried out in person at the Lakeshore Foundation in November 2023. One Lakeshore Foundation senior staff member was unable to attend the focus group session, so they completed a follow-up, one-on-one, semistructured interview online in December 2023. Interviews and focus groups were completed with 79% of program participants and relevant staff and volunteers.

#### Data Analysis and Rigor

Survey data and participant characteristics are summarized using descriptive statistics. Means and SDs are reported for the continuous variables (ie, age and AIM, IAM, and FIM surveys), while categorical variables (ie, race and sex) are presented as frequencies and percentages.

All qualitative data were coded and organized using NVivo 14 (Lumivero). In the first stage of analysis, the Zoom recordings were transcribed verbatim using a professional transcription service (Landmark Associates, Inc), with time stamps at the beginning of each response and speakers labeled as interviewer or interviewee to ensure clarity and anonymity of the content. The first author became familiar with the data by listening to the audio recordings and repeatedly reading the transcripts. The data were then analyzed using a deductive content analysis approach, following the 3-phase guide outlined by Elo and Kyngäs [41], which includes preparation, organization, and reporting. During the preparation phase, it was decided that sentences, phrases, and themes from the transcripts would serve as the units of analysis to capture both detailed content and broader thematic patterns. Both manifest (explicit, surface-level content) and latent (underlying meanings) content were coded, allowing for a more nuanced understanding of factors influencing implementation. A deductive approach was chosen to align the identified themes with the CFIR domains and constructs, facilitating a theory-driven analysis of implementation factors.

In phase 2 of the qualitative analysis, a structured categorization matrix based on the 5 CFIR domains and 39 constructs was developed to guide organization and interpretation of themes. The first author then reviewed the data for content and coding based on correspondence with the predetermined domains and constructs. In phase 3, results were synthesized by identifying the most frequently reported themes. To ensure accurate alignment with CFIR constructs, the first and second authors reviewed and refined the synthesized results, confirming that each theme was appropriately categorized and all relevant data were considered. Integration occurred during the interpretation phase, where the survey results were used to provide supplementary context and illustration for the core qualitative themes. In this analysis, the main findings remain rooted in the qualitative evidence and are organized by facilitators and barriers, specific CFIR domains, and subthemes that correspond to the CFIR constructs [29].

To ensure quality and rigor, the evaluative markers coherence, transparency, and substantive contribution were selected from Smith and Caddick’s [42] ongoing list and can be found in Table 1. At the time of data collection, the first author (WNN) was a predoctoral student in health behavior with formal training in qualitative research methods. With nearly a decade of experience working with individuals with chronic health conditions and no prior involvement in the study’s program delivery portion, she brought an outsider perspective. The second author (LAM) has been involved in several qualitative studies related to physical activity and disability and served as a “critical friend” throughout the process. This role involved reviewing the first author’s interpretations of the data and providing feedback on the accuracy and consistency of the coding and categorization process. Rather than reach an agreement, this collaborative, reflective approach was intended to generate nuanced and multifaceted interpretations of meaning.

**Table 1.** Evaluative markers chosen for quality and rigor.

Marker	Definition	Application
Coherence	The way that different parts of the interpretation create a complete and meaningful picture.	Demonstrated through the alignment of the research questions, methodology, and validated theoretical framework to guide the evaluation.
Transparency	Honesty about the research process.	Maintenance of an audit trail through detailed notes and critical friend throughout analytical process.
Substantive contribution	The extent to which a study makes a meaningful impact on our understanding of what is being researched theoretically, conceptually, practically, or methodologically.	Offers a theoretical understanding of factors influencing the implementation of technology-driven community programs.

## Results

### Implementation Surveys

All administrators, program staff, and volunteers involved in implementing the LOF and VR programs (n=9, 100%) completed the surveys. Results from the AIM, IAM, and FIM surveys are provided in Table 2. Overall, both programs attained high ratings across all 3 domains. However, one-third of respondents (n=3, 33.3%) rated the VR program’s feasibility as medium (FIM score <15) rather than high.

**Table 2.** Acceptability, appropriateness, and feasibility measure results.

	LOF^a^ program, mean (SD)	VR^b^ program, mean (SD)
AIM^c^	16.89 (1.62)	17.56 (2.74)
IAM^d^	16.33 (1.58)	16.56 (2.70)
FIM^e^	16.78 (1.64)	16.22 (2.86)

a LOF: Lakeshore Online Fitness

b VR: virtual reality

c AIM: Acceptability of Intervention Measure

d IAM: Intervention Appropriateness Measure

e FIM: Feasibility of Intervention Measure

### Interview and Focus Group Results

A total of 15 individuals took part in the interviews (mean 41.3 min, SD 16.1 min; range 24‐63 min) or focus groups (mean 49.3 min, SD 7.1 min), including 8 of 10 program participants, 1 recreation center staff member, 2 community volunteers, 2 fitness instructors, and 2 administrators [1]. Interview participants (n=8) were primarily Black or African American (n=7, 87.5%) and female (n=5, 62.5%) participants with a mean age of 65.0 (SD 14.0; range 44‐86) years. The majority of interview participants had hypertension (n=6, 75%) and arthritis (n=4, 50%), with 37.5% having both concurrently. Focus group participants were primarily White (5 /7, 71.4%) and male (4/7, 57.1%) individuals; age was not collected. Program participants completed 78.3% and 85.4% of LOF and VR sessions, respectively.

**Figure 3.**
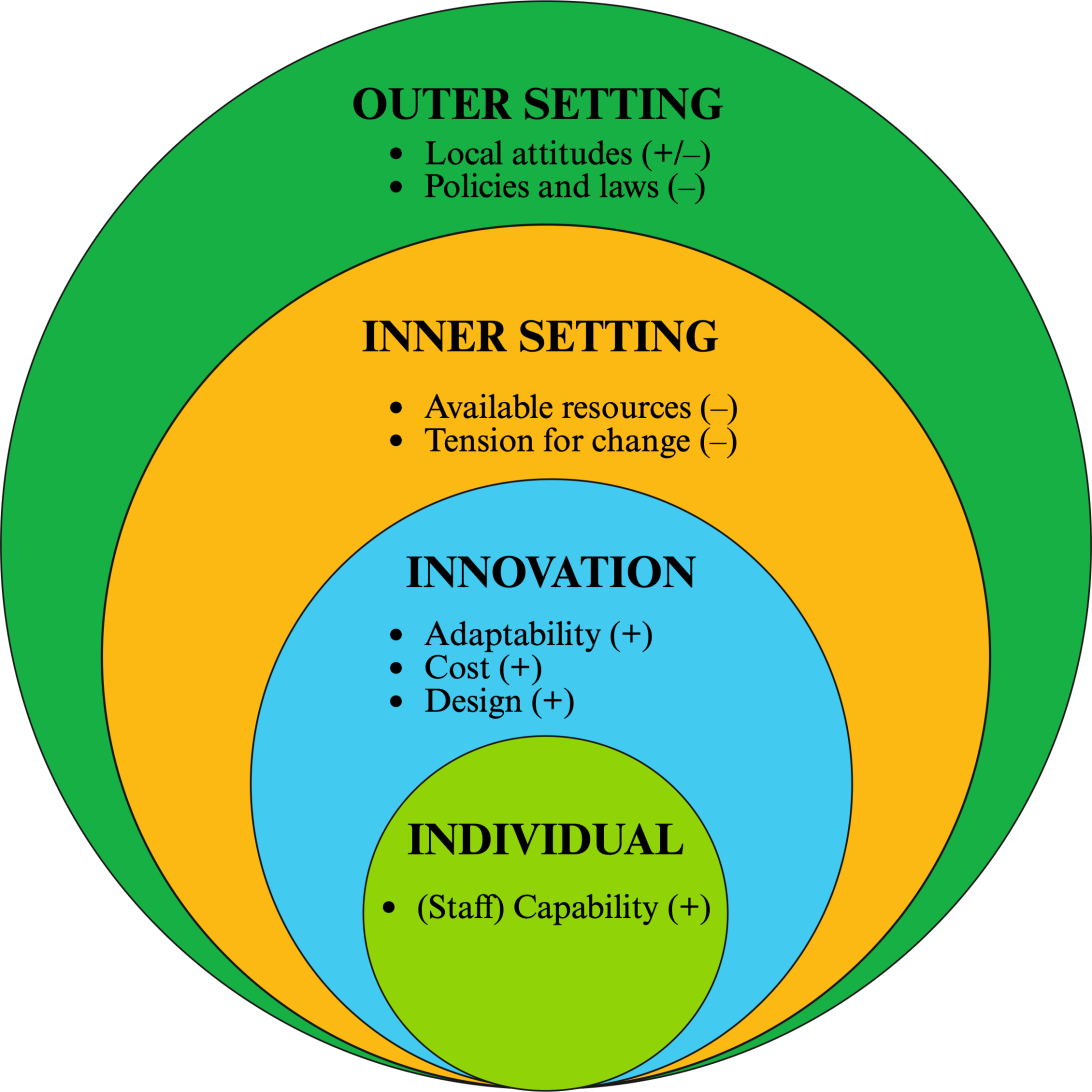
Implementation barriers and facilitators coded into Consolidated Framework for Implementation Research (CFIR) domains and constructs. “–“ indicates barriers, “+” indicates facilitators. Created in BioRender [43].

Qualitative data analysis identified 4 overarching domains and 8 constructs that represented key facilitators and barriers to implementing the LOF and VR programs. The results mapped to CFIR domains and constructs are depicted in Figure 3.

### Key Facilitators to LOF or VR Implementation

Six subthemes based on 5 CFIR constructs were identified as facilitators to program implementation. These constructs belonged to 3 CFIR domains (“innovation,” “outer setting,” and “individuals”) and are described in more detail under the “Innovation,” “Outer Setting,” and “Individuals” subsections below.

#### Innovation

##### Overview

The innovation domain comprises features of an intervention that might influence implementation. Key facilitators related to program characteristics centered on the adaptability, cost, design quality, and packaging constructs. Implementation team members praised both the LOF and VR programs for their flexibility, allowing for modifications to accommodate diverse physical abilities. The low cost of equipment and free access at the community center made both programs accessible and affordable, while the socially interactive and engaging design enhanced motivation and enjoyment for program recipients.

##### Flexible LOF Exercises (Adaptability)

All program recipients and deliverers noted the flexibility of the LOF program for participants with diverse physical abilities, such as low mobility, dexterity limitations, low vision, or stroke-related impairments. Participants with dexterity limitations mentioned the ease of modifying the LOF program components to facilitate engagement, “Once I could pick up the weights, when the resistance ball isn’t—none of it bothered me, it was just putting it in my hand” (program recipient 04). Instructors adjusted exercises to participants’ needs without full information on their abilities but noted that experience was essential for making these on-the-fly adaptations.

##### VR Program Technology Considerations (Adaptability)

Recreation center staff and volunteers emphasized the importance of the VR program’s adaptability to diverse settings, such as standing versus seated sessions to support group activity and ensure participant safety:

*I think if they were standing up, they would likely free roam. They’re in their virtual world, and they can’t see where they’re going or what they’re doing. When you start moving, you just movin’. They might be bumping into each other. I think sitting was the perfect thing to stay in the circle and not bump each other. I think that was good*.

[Community volunteer 01]

Although some participants initially struggled with dexterity, this issue was resolved quickly by the research staff, who modified the VR equipment for one-handed use.

##### Program Access and Affordability (Cost)

Program deliverers felt that the LOF program’s low-cost exercise equipment, such as balls, bands, and lightweight dumbbells, would make the program relatively simple to replicate in other locations:

*In theory, the way this works is we’re bringing a lot of the expense to them. We’re bringing the TV. We’re trying to cut away as much of the red tape as we can. What we really need is for them to say, yes, here’s a room. Here’s somebody to turn on the TV. Here’s somebody who can hover in there to help with stuff, but you guys do the hard part of buying the TV and having the expertise to teach the class. It’s just [a] partnership*.

[Administrator 01]

Program recipients appreciated that both programs were free and available at the community center, citing affordability and ease of access as key facilitators of engagement. They acknowledged that many older adults and people with chronic health conditions in their community lacked transportation to attend paid physical activity programs, but that, “…they would be happy to do exercise like that, long as they don’t have to leave the building’’ (program recipient 09).

##### Socially Interactive and Engaging Design (Design)

This subtheme pertains to participant perceptions of the quality of the materials associated with the LOF and VR programs. Program recipients valued the socially supported, group-based nature of the LOF program, which enhanced motivation. “We did those stretch band things. We did the balls and the dumbbells…I liked it. I liked being on the equipment” (program recipient 02). They found the competitive and interactive elements of the VR program particularly motivating, as these elements allowed them to compete against others or themselves, “We had a competition going together shooting games and stuff like that, bowling, and stuff like that” (program recipient 03). These program components made physical activity more enjoyable and immersive:

*I like the [VR] headset because you get the feeling of really being in there. That’s the part I really like. The sceneries are so beautiful. You really did, you felt like you had actually stepped into a space age, or a tennis court or baseball diamond*.

[Program recipient 08]

### Outer Setting: Social and Community Engagement (Local Attitudes)

This subtheme reflects the degree to which sociocultural values (eg, shared responsibility in helping recipients) and beliefs (eg, convictions about the worthiness of recipients) encourage the outer setting (eg, the city the program was implemented in) to support implementation and/or delivery of the intervention.” [30,31] Those involved in program implementation and delivery felt that, “Having a community champion like the assistant mayor*”* (administrator 01) was crucial to the program’s success, as their advocacy facilitated connections with community leaders and strengthened local engagement. Positive reactions from program recipients further affirmed the programs’ value and energized program deliverers. These groups noted that this enthusiasm made the program more enjoyable for the staff and was a key indicator of its success:

*Just seeing them enjoying made me feel good, because they [were] enjoying something. It’s not like they [were] forced to do it or “Okay, we’re in this program here at the rec, so this is part of it. We have to do it.” “No, this is a program that I enjoy. I’m glad when it start[s]. I hate when it end[s]. We just want to have fun.” They all got along. That was a good thing*.

[Community volunteer 01]

*...it was fun to coach, because you could see them move and you could see ’em doing things and it seemed like they really enjoyed it and then when it ended, they would clap and say thank you, and all that and give you that reassurance*.

[Fitness instructor 01]

### Individuals: Staff Training and Role Clarity (Capability)

Effective implementation required clear role distribution and adequate staffing to assist participants, manage technology, and troubleshoot issues onsite. Program deliverers felt that staff with strong interpersonal and problem-solving skills and at least some knowledge of exercise were beneficial to LOF and VR program success:

*So, ideally, it would be someone with fitness experience…[but] minimally, it has to be, in my opinion, someone who is consistent, dependable, and someone that we could train*.

[Administrator 02]

*It was a very easy setup and once the class was going, you were hands off. If someone didn’t really seem like they knew what they were doing, you would try and mimic what the instructor was doing. I don’t really feel like there was too much training needed for that portion of it*.

[Recreation center staff 01]

### Key Barriers to LOF or VR Implementation

There were 4 barriers identified from the interviews and focus groups, with subthemes divided evenly between the inner and outer setting domains.

#### Inner Setting

##### Overview

Barriers to LOF and VR program implementation within the inner setting domain (eg, the organization in which the intervention is being implemented) [30,31] centered on the available resources and tension for change constructs. The LOF and VR programs at the recreation center faced technical and spatial challenges, and program recipients expressed dissatisfaction with the limited accessible physical activity options in their area, highlighting the need for sustainable community resources to address underlying infrastructure deficits.

##### Technology and Space Challenges (Available Resources)

This subtheme reflects “the degree to which resources are available to implement and deliver the intervention” [30,31] Addressing technical and spatial barriers, including technology setup and space constraints, was essential to successful implementation of the LOF and VR programs at the recreation center. Stable Wi-Fi, reliable equipment, and adequate space were critical to overcoming these challenges and ensuring successful program delivery. Program users reported minimal technical issues:

*The only [LOF program] technical issue I think we had—well, then it wasn't an issue—was that we could not hear what she was sayin', but we could see what she was doin', so we still continued because we could see her doin’ the exercises*.

[Program recipient 05]

*One day the [VR program] computer wasn’t acting right for some. … [But] we never had to miss a day because of the technical difficulties*.

[Program recipient 08]

However, program providers felt that LOF program delivery was complicated by spatial constraints at the recreation center:

*I think the biggest complaint with the [LOF] class was we just didn’t have enough space… Sometimes we had 10 people, and so we had all the chairs. It was like they couldn’t fully put out their arms, or [they’d] be running into each other and stuff like that. … I feel like just the size of the room was the biggest issue*.

[Recreation center staff 01]

*You could see 'em moving and everything, but you weren't able to read people’s eyes, you saw gross movements, but you didn't see the finer movements*.

[Fitness instructor 01]

LOF instructors noted that participant monitoring could be enhanced with larger screens and/or additional cameras, “…so you can see people a little bit closer, ’cause we were at a distance, and you really couldn’t see their faces” (fitness instructor 01). Instructors also emphasized the importance of standardized and simplified technological setups on their end to minimize the burden of adjusting to new rooms, equipment, and lighting conditions.

##### Need for Infrastructure and Resource Support (Tension for Change)

This subtheme aligns with the CFIR construct “tension for change,” where implementation team member recognition of significant gaps creates momentum for implementation efforts [30,31]. Program recipients expressed a strong desire for more accessible physical activity options in their area, noting that the absence of such resources created what they perceived as an urgent community need: “There aren’t no other [free physical activity opportunities] here...People, they go out down there to the community store and buy ’em a can of beer or a pack of cigarettes*”* (program recipient 04). Participants highlighted both the immediate value of the LOF and VR programs, “Well, me and my other two siblings, we got high blood pressure, so we need somethin’ to motivate us” (program recipient 02), and emphasized the need for additional community resources to support these programs long-term:

...*Cause we don’t have nothin’ there. I needed it. ...Those people need it. They really need somethin’ like that…*

[Program recipient 09]

The emphasis on sustainability concerns further reflected program recipients’ insight that temporary interventions, while beneficial, would not address the fundamental infrastructure deficits underlying their community’s physical activity challenges.

### Outer Setting

Key barriers to LOF and VR program implementation within the outer setting domain centered on the local attitudes and policies and laws constructs. Resistance to new technology and sustainability challenges were identified as barriers to implementing and sustaining the LOF and VR programs.

#### Resistance to New Technology and Approaches (Local Attitudes**)**

Program recipients, administrators, recreation center staff, and volunteers all mentioned resistance to adopting new technology and program approaches. Some program recipients expressed concerns that others in the community might resist the VR program due to discomfort with wearing headsets, influenced by cultural and personal preferences: “...I don’t think they would like it ’cause just puttin’ stuff on their face…*”* (program recipient 09). Similarly, recreation center administration initially doubted participants’ willingness to engage with the LOF and VR programs, reflecting skepticism about the programs’ acceptance and feasibility:

*When we said, well, how about this group take the class, [senior staff] at the rec center were like, y’all are out of your minds. They are never going to participate in this*

[Administrator 01]

#### Sustainability Challenges (Policies and Laws)

This subtheme reflects the “degree to which legislation, regulations, professional group guidelines and recommendations, or accreditation standards support implementation and/or delivery of an intervention” [30,31]. One administrator felt that relying solely on grant funding made long-term sustainability of the LOF program uncertain, especially without a financial model for continued implementation, “So how do we deliver it when it’s not through a grant, and how do we try to align as closely to the way that we would onboard members now, but on the online, which is different*”* (administrator 02). This administrator also noted liability and membership issues, including the need for waivers and consistent liability protocols, as barriers to continuing the LOF program at the community center, “...Because we can’t deliver any product without some kinda safeguard set up...and I don’t believe we ever figured that out*”* (administrator 02). Similarly, program deliverers expressed concerns about the VR program’s long-term feasibility due to equipment maintenance, cultural unfamiliarity, and staff burden—factors that align with the relatively lower feasibility scores reported in the FIM survey.

## Discussion

### Key Findings and Contributions

This study applied the CFIR to explore barriers and facilitators influencing implementation of two technology-driven group exercise programs—an online platform (LOF) and VR gaming—delivered to predominantly African American older adults living in an underserved community. Through semistructured interviews, focus groups, and implementation surveys, we identified factors across 4 CFIR domains (innovation, outer setting, inner setting, and individuals) that collectively influenced the programs’ uptake, delivery, and sustainability. Our findings highlight that successful implementation was shaped by a combination of program adaptability, affordability, social engagement, and community support, while sustainability and resource constraints emerged as persistent barriers.

A key contribution of this study lies in demonstrating how technology-enabled interventions can create structured exercise opportunities in community centers that previously lacked formal fitness programming. Unlike settings where technology supplements existing exercise classes, the recreation center in this study had no structured physical activity programs prior to implementation. Technology served as the catalyst for introducing accessible, engaging, and adaptable exercise options, addressing a critical gap in underserved communities where traditional programs are often unavailable due to resource limitations. This approach aligns with growing evidence that technology-based interventions can overcome environmental and logistical barriers by delivering scalable, customizable programs that promote physical activity and social engagement among older adults and individuals with disabilities [18-20]. By leveraging online platforms and VR gaming, the programs provided not only exercise opportunities but also novel experiences that enhanced enjoyment and motivation—factors shown to improve adherence in populations facing mobility and transportation challenges [15,23,26].

### Facilitators of Implementation

Adaptability emerged as a key facilitator across both programs. The ability to adjust exercises and technology to accommodate diverse physical abilities allowed for broad participation, even among individuals with mobility limitations and dexterity challenges. These findings align with previous research highlighting the importance of customizable interventions that support engagement among populations with heterogeneous physical limitations [44,45]. Affordability and ease of access were also critical enablers, extending beyond equipment costs to include transportation and membership fees, which are common deterrents among people with disabilities [1]. By eliminating these costs, the programs created equitable access to structured exercise opportunities, reinforcing prior evidence that cost-sensitive solutions are vital for program sustainability in resource-constrained settings [46].

The socially interactive and gamified design of both programs further enhanced motivation and enjoyment. LOF’s group-based format fostered camaraderie, while the VR program’s competitive elements introduced novelty and fun, supporting the value of incorporating social and gamified components into technology-based group physical activity programs for older adults and people with disabilities to sustain engagement [47]. Future implementation of technology-driven exercise interventions should leverage these design principles to strengthen both physical and psychosocial outcomes in this population.

Finally, community support amplified program success. Advocacy from community champions (eg, city leaders), enthusiastic endorsement from community volunteers and recreation center administrators, and visible program recipient enthusiasm strengthened buy-in among staff and volunteers, creating a sense of shared ownership. This is consistent with previous research emphasizing the role of community advocacy in successful implementation of health promotion programs in underserved areas [45] and suggests that early identification of community champions should be integral to implementation planning.

### Barriers to Implementation

Despite these strengths, several barriers impeded implementation. Technology and space limitations were the most immediate obstacles. Unstable Wi-Fi, suboptimal camera setups, and inadequate physical space complicated LOF program delivery and monitoring, while the VR sessions required careful configuration to ensure safety, highlighting the need for robust infrastructure and standardized protocols [45,46,48]. Resistance to new technology reflected cultural and personal discomfort, particularly with VR headsets, and skepticism among staff about program feasibility. While program recipients spoke highly of the VR program, initial hesitancy underscores the importance of tailored program orientation and gradual exposure to technology to build confidence and acceptance [45,49,50].

Additionally, sustainability challenges stemming from reliance on grant funding and unresolved liability protocols raised concerns about program continuity. Administrations cited liability and membership protocols as unresolved issues for the LOF program, while survey responses from program deliverers reflected reservations about the VR program’s feasibility. This underscores the need for alternative funding strategies (eg, partnerships with local health departments and sliding-scale fees), infrastructure development, and policy alignment to support long-term program continuation. Without such planning, even well-received programs risk discontinuation once initial funding ends.

### Implications

Our findings suggest that technology-driven exercise programs can reduce health disparities in underserved communities when they are adaptable, affordable, socially engaging, and supported by community champions. However, implementation efforts must anticipate and address resource constraints, technology resistance, and sustainability challenges. Multilevel implementation strategies, including investment in digital infrastructure, community advocacy, targeted orientation to reduce technology hesitancy, and sustainable financing models, are essential to scale and sustain such interventions. Future research should explore strategies for scaling these programs, such as partnerships with local organizations, integration into existing community services, or development of cost-sharing models. Programs should engage community champions from inception, build standardized technical protocols, and test these programs across diverse geographic and demographic contexts.

### Strengths and Limitations

Study strengths include its use of a validated implementation framework (CFIR), integration of multiple implementation team member perspectives, and use of both qualitative and quantitative methods to identify barriers and facilitators to program implementation. However, the current study has limitations that warrant consideration. Our sample, though achieving 79% participation among relevant implementation team members, was relatively small and drawn from a single, predominantly African American community, which may limit generalizability. Additionally, racial distribution differed between data collection formats: interview participants were predominantly African American, while focus group participants were primarily White. This imbalance may have influenced the range of perspectives captured. Implementation team member perspectives were collected several months after program delivery, which may not have captured immediate perspectives of program sustainability or technology acceptance. Nonetheless, the rich qualitative insights underscore the value of incorporating ongoing implementation team member evaluation and feedback into program design.

### Conclusions

Technology-enhanced group exercise interventions hold considerable promise for reducing physical inactivity among underserved older adults and people with disabilities. The results of our CFIR-guided analysis suggest that, while adaptability, low cost, and social engagement facilitated uptake, the LOF and VR programs presented technical, infrastructural, and policy-related barriers. The findings of this study contribute to the growing literature on technology-enabled health interventions for aging populations and people with disabilities and offer insights into implementation strategies in underserved, resource-limited settings.

## Supplementary material

10.2196/79598 Multimedia Appendix 1 Lakeshore Online Fitness or Get Active with Virtual Reality intervention participant interview guide.

10.2196/79598 Multimedia Appendix 2 Lakeshore Online Fitness or Get Active with Virtual Reality intervention focus group guide.

## Abbreviations

AIM: Acceptability of Intervention Measure
CFIR: Consolidated Framework for Implementation Research
FIM: Feasibility of Intervention Measure
IAM: Intervention Appropriateness Measure
LOF: Lakeshore Online Fitness
VR: virtual reality
