# Supplementary material for: Technology-Driven Group Exercise Program Implementation in an Underserved Community: Multimethod Retrospective Evaluation Study
Source: JMIR Rehabil Assist Technol. 2026 Mar 24;13:e79598. doi: 10.2196/79598 (PMC13012231; doi:10.2196/79598)
Supplement: Multimedia Appendix 1 [file rehab-v13-e79598-s001.docx]

Multimedia Appendix. Lakeshore Online Fitness/Get Active with Virtual Reality Intervention Participant Interview Guide

**Grand Tour Questions**

1. First, can you tell me about yourself?
   - General life - work/family/hobbies
   - Living situation, years as Tarrant resident?
   - Years of membership at community center?
2. Can you tell me about your experiences with physical activity?
   - What has helped you be physically active?
   - What has made it difficult for you to be physically active?
   - Does where you live affect your participation in physical activity?
   - What were your reason(s) for participation in the program?
   - Would you continue to participate in the online fitness program if it were available? In the virtual reality program?
3. What are your opinions about using technology for the purpose of keeping fit and healthy?
   - Do you have any concerns about using virtual reality to keep fit and healthy?

**Knowledge and beliefs about the intervention**

1. Can you tell me about your experience with the online fitness program being used at the community center?
   1. What did you like most about the online fitness program? What did you like the least about the program?
   2. What barriers did you face to participating in the program?
2. Can you tell me about your experience with the virtual reality program being used at the community center?
   1. What did you like most about the virtual reality program? What did you like the least about the program?
   2. What barriers did you face to participating in the program?
3. In your opinion, is there a strong need for the online fitness and virtual reality programs in your community?
4. How suitable/acceptable/appropriate are the online fitness and virtual reality programs for the individuals served by the community center?
   1. In what ways do these programs meet their needs (e.g., improved access to exercise, help with self-management, reduced travel time and expense)?
5. How well do you think the technology-based physical activity programs address social isolation and loneliness?

**Relative advantage**

1. Is there another physical activity program in your community that you would rather participate in?
   1. Can you describe that program?
   2. Why would you prefer the alternative?
   3. How do the online fitness and virtual reality programs compare to that physical activity program?

**Design quality and features**

1. What did you think about the quality of the online fitness program (ie, class variety, instructor teaching style, group physical activity)?
   1. What did you think about the provided equipment for the online fitness program (ie, TV, exercise equipment including resistance bands, balls, and hand weights)?
   2. Were there any technical issues while participating in the online fitness program?
   3. What did you think about the duration and intensity of the online fitness classes?
2. What did you think about the quality of the virtual reality program (ie, variety, type, of games?
   1. Did you feel part of the virtual environment?
   2. What did you think you about playing in the same physical space as others?
   3. Were there any technical issues while participating in the virtual reality program?
   4. Did it take long to figure out how the virtual reality system works? What took the most time to figure out? Was the trial sufficient to understand the virtual reality system?

**Adaptability**

1. What other changes do you think we will need to make to the online fitness program so it will work effectively in your community?
2. What other changes do you think we will need to make to the virtual reality program so it will work effectively in your community?
3. Is there anything else you would like to say about the online fitness and virtual reality programs that has not been covered?

This is a Multimedia Appendix to a full manuscript published in the JMIR Rehabil. Assist. Technol. For full copyright and citation information see http://dx.doi.org/10.2196/jmir.xxxx
