# Supplementary material for: Technology-Driven Group Exercise Program Implementation in an Underserved Community: Multimethod Retrospective Evaluation Study
Source: JMIR Rehabil Assist Technol. 2026 Mar 24;13:e79598. doi: 10.2196/79598 (PMC13012231; doi:10.2196/79598)
Supplement: Multimedia Appendix 2 [file rehab-v13-e79598-s002.docx]

Multimedia Appendix. Lakeshore Online Fitness/Get Active with Virtual Reality Intervention Focus Group Guide

1. [Senior staff only] What are your thoughts about the process of setting up the contracts to implement the programs?
   1. Are there parts of the process that should be changed?
   2. Which parts should not be changed?

**Lakeshore Online Fitness**

1. What did you think about the quality of the Lakeshore Online Fitness program?
   1. Are there components of the program that should be changed?
   2. Which components should not be changed?
2. What did you think about your time and energy commitment required to implement the Lakeshore Online Fitness program?
3. Is there a need for the Lakeshore Online Fitness program at the community center? Why or why not?
4. How well does the Lakeshore Online Fitness program fit with existing work processes/operations (ie, staffing, facility use, timing/scheduling) at the community center?
5. Please describe any barriers to continuing the Lakeshore Online Fitness program.
6. Do you feel that you have sufficient resources (ie, space, time, staffing, funding, etc.) to continue administering/delivering the Lakeshore Online Fitness program?
   1. If yes, what resources do you have?
   2. If no, what resources would be needed to continue delivering the program?
7. Please describe how the Lakeshore Online Fitness program could be integrated into your current processes/operations.
8. What type of support from each party (Lakeshore Foundation, Tarrant Parks and Recreation Department, City of Tarrant) do you feel is needed to continue the program?
9. What adjustments do you think should be made to the Lakeshore Online Fitness program before making it available to other communities?

**Virtual Reality Program**

1. What did you think about the quality of the Virtual Reality program?
   1. Are there components of the program that should be changed?
   2. Which components should not be changed?
2. What did you think about your time and energy commitment required to implement the Virtual Reality program?
3. Is there a need for the Virtual Reality program at the community center? Why or why not?
4. How well does the Virtual Reality program fit with existing work processes/operations (i.e., staffing, facility use, timing/scheduling) at the community center?
5. Please describe any barriers to continuing the Virtual Reality program.
6. Do you feel that you have sufficient resources (i.e., space, time, staffing, funding, etc.) to continue administering/delivering the Virtual Reality program?
   1. If yes, what resources do you have?
   2. If no, what resources would be needed to continue delivering the program?
7. Please describe how the Virtual Reality program could be integrated into your current processes/operations?
8. What type of support would be needed to continue the Virtual Reality program?
9. What adjustments should be made to the Virtual Reality program before making it available to other communities?

This is a Multimedia Appendix to a full manuscript published in the JMIR Rehabil. Assist. Technol. For full copyright and citation information see http://dx.doi.org/10.2196/jmir.xxxx
